# Predicting Overall Survival in Patients with Male Breast Cancer: Nomogram Development and External Validation Study

**DOI:** 10.2196/54625

**Published:** 2025-03-04

**Authors:** Wen-Zhen Tang, Shu-Tian Mo, Yuan-Xi Xie, Tian-Fu Wei, Guo-Lian Chen, Yan-Juan Teng, Kui Jia

**Affiliations:** 1 Department of Hepatobiliary Surgery, The First Affiliated Hospital of Guangxi Medical University, Nanning, China; 2 Department of Central Sterile Supply, The First Affiliated Hospital of Guangxi Medical University, Nanning, China; 3 Department of Gastrointestinal Surgery, The First Affiliated Hospital of Guangxi Medical University, No 6 Shuangyong Road, Nanning, 530021, China, +86 0771-12580-6; 4 Department of Medical Oncology, The First Affiliated Hospital of Guangxi Medical University, Nanning, China

**Keywords:** male breast cancer, specific survival, prediction model, nomogram, Surveillance, Epidemiology, and End Results database, SEER database

## Abstract

**Background:**

Male breast cancer (MBC) is an uncommon disease. Few studies have discussed the prognosis of MBC due to its rarity.

**Objective:**

This study aimed to develop a nomogram to predict the overall survival of patients with MBC and externally validate it using cases from China.

**Methods:**

Based on the Surveillance, Epidemiology, and End Results (SEER) database, male patients who were diagnosed with breast cancer between January 2010, and December 2015, were enrolled. These patients were randomly assigned to either a training set (n=1610) or a validation set (n=713) in a 7:3 ratio. Additionally, 22 MBC cases diagnosed at the First Affiliated Hospital of Guangxi Medical University between January 2013 and June 2021 were used for external validation, with the follow-up endpoint being June 10, 2023. Cox regression analysis was performed to identify significant risk variables and construct a nomogram to predict the overall survival of patients with MBC. Information collected from the test set was applied to validate the model. The concordance index (C-index), receiver operating characteristic (ROC) curve, decision curve analysis (DCA), and a Kaplan-Meier survival curve were used to evaluate the accuracy and reliability of the model.

**Results:**

A total of 2301 patients with MBC in the SEER database and 22 patients with MBC from the study hospital were included. The predictive model included 7 variables: age (hazard ratio [HR] 1.89, 95% CI 1.50‐2.38), surgery (HR 0.38, 95% CI 0.29‐0.51), marital status (HR 0.75, 95% CI 0.63‐0.89), tumor stage (HR 1.17, 95% CI 1.05‐1.29), clinical stage (HR 1.41, 95% CI 1.15‐1.74), chemotherapy (HR 0.62, 95% CI 0.50‐0.75), and HER2 status (HR 2.68, 95% CI 1.20‐5.98). The C-index was 0.72, 0.747, and 0.981 in the training set, internal validation set, and external validation set, respectively. The nomogram showed accurate calibration, and the ROC curve confirmed the advantage of the model in clinical validity. The DCA analysis indicated that the model had good clinical applicability. Furthermore, the nomogram classification allowed for more accurate differentiation of risk subgroups, and patients with low-risk MBC demonstrated substantially improved survival outcomes compared with medium- and high-risk patients (*P*<.001).

**Conclusions:**

A survival prognosis prediction nomogram with 7 variables for patients with MBC was constructed in this study. The model can predict the survival outcome of these patients and provide a scientific basis for clinical diagnosis and treatment.

## Introduction

Male breast cancer (MBC) is an infrequent type of malignancy [1,2]. The incidence of MBC accounts for less than 1% of all breast cancer (BC) instances, and MBC accounts for 0.31% of all BC cases in China [3-5]. The incidence of MBC varies by region and ethnicity, with higher rates observed in Africa, North America, and Australia, and the lowest rates are observed in Asia [6]. In China, there are only 4 cases of MBC per million people, but this figure has been increasing gradually in recent years [6]. Due to the low incidence of MBC, current research on BC primarily focuses on female patients [7]. Therefore, the current treatment for MBC is based on the guidelines for treating female BC [8]. However, MBC possesses unique tumor, molecular, and clinicopathological characteristics, and no consensus has been established regarding its diagnosis, treatment, and assessment of prognostic risk factors. A previous study revealed that the median age at diagnosis for BC in men is 67 years old, which is 5‐10 years later than that in women [9]. Despite this, the overall survival of MBC is significantly lower than that of female BC, largely due to late diagnosis [10].

The TNM (tumor, extent of spread to the lymph nodes, and presence of metastasis) staging system is the most commonly used clinical instrument to evaluate the prognosis of individuals with cancer [11-13]. However, in MBC, the limited amount of breast tissue and the frequent involvement of the chest wall at early stages reduce the prognostic value of TNM staging [14]. Many studies have demonstrated that factors such as age, tumor type, and other factors significantly influence the prognosis of BC [15,16]. Compared to using the clinical stage alone, comprehensive multivariate models can provide numerical estimates of practice-specific risk and the accuracy of prognostic predictions for patients with cancer [17]. Therefore, various clinical medical records need to be combined to construct a prognostic model for MBC, thus enabling a more accurate judgment of the prognosis of patients and an accurate, individual evaluation of the prognosis of patients.

Current clinical approaches for constructing risk prediction models include the nomogram, a scoring system, and other methods, which can serve as a guide for clinical decision-making and individualized treatment [18-20]. The nomogram, as a straightforward and intuitive prediction tool with strong predictive ability, has the advantages of accurate predictive ability and calibration ability, and it has been widely used in prognosis research [21-23].

This study aimed to identify the prognostic indicators of patients with MBC by using the Surveillance, Epidemiology, and End Results (SEER) database; establish a predictive model on the basis of the independent predictors of overall survival; and internally and externally validate the model to guide clinical staff in evaluating the prognosis of patients more accurately and formulating more personalized diagnosis and treatment plans. We present the study in accordance with the TRIPOD reporting checklist.

## Methods

### Data Sources

The SEER database collects information on new cancer cases and survival rates from 18 population-based cancer registries, which currently cover approximately 30% of the US population [22]. Clinical data on male patients with pathologically confirmed BC from 2010 to 2015 were gathered using the SEER database to establish a training set and an internal validation set. Data from patients with MBC admitted to the First Affiliated Hospital of Guangxi Medical University between 2013 and 2021 were used for the external validation set of the model. Clinical data for MBC were retrospectively collected from the hospital database, and follow-up information was obtained through telephone interviews. Patients with missing follow-up data or other essential clinical information were excluded.

### Patient Inclusion and Exclusion Criteria

The criteria for patient inclusion were as follows: (1) male patients; (2) an *International Classification of Diseases for Oncology, Third Revision* code; (3) breast as the primary site; and (4) complete survival data. The exclusion criteria were (1) missing clinical information, including TNM staging and tumor laterality; (2) unknown demographic characteristics, such as age at diagnosis and marital status; and (3) instances without records of follow-up (0-month survival time code). The enrolled patients were randomly assigned in a 7:3 ratio to two sets: a training set and an internal validation set. The training set was used to develop the prediction model, and the internal validation set was used for internal validation. The data obtained at the hospital were applied for external validation.

### Variable Selection

The outcome variable in this study was overall survival. The selection of predictor variables was informed by previous reports in the literature. The variables collected included year of diagnosis, age, marital status, pathological grade, breast subtype, estrogen receptor, progesterone receptor, human epidermal growth factor receptor 2 (HER2), American Joint Committee on Cancer stage, chemotherapy, surgery, radiotherapy, duration of follow-up, and death.

### Follow-Up of Patients

Male patients with BC were followed up by telephone in the hospital, and the follow-up ended on June 10, 2023. The index used for follow-up was overall survival time, with the outcome event being mortality.

### Statistical Analysis

Data analysis was conducted using the R software version 4.1.1 (IBM Corp). Percentages were used to represent categorical variables, and the *χ*^2^ test or Fisher exact test was used to compare the baseline characteristics of the training set, internal validation set, and external validation set. The Kaplan-Meier model was applied to describe the overall survival curve, and the log-rank test was used to evaluate the disparities in survival among various subgroups of each variable. First, variables that had a significance value of *P*<.05 in the univariate analysis were chosen to be incorporated into the multivariate Cox proportional hazard model to obtain variables affecting the survival of patients with MBC. Second, stepwise regression was performed based on the Akaike information criterion. The nomogram prediction model was constructed using R software (via the *rms* and *survival* R packages) to assess the influence of risk factors on the overall survival of patients with MBC. Predictions were made for the 1-, 3-, and 5-year overall survival rates of patients with MBC by constructing the nomogram.

The performance of the nomogram was evaluated through internal and external validations. Bootstrapping was used to perform 1000 instances of resampling to internally validate the predictive performance of the nomogram to ensure the stability and reliability of the model’s performance. The discrimination of the nomogram was assessed using the Concordance index (C-index) and receiver operating characteristic (ROC) curve. A calibration curve was created to assess the degree of calibration of the nomogram to ensure its accuracy and reliability. Furthermore, decision curve analysis (DCA) was conducted using *ggDCA* in the R package, to evaluate the clinical utility and application value of the nomogram. Finally, X-tile software (version 3.6.1, Yale University School of Medicine) was used for risk stratification on the basis of the total score of the nomogram for each individual. An α level of .05 was used.

### Ethical Considerations

The data used in this study were extracted from a publicly accessible SEER database. This study was reviewed and approved by the Medical Ethics Committee of the First Affiliated Hospital of Guangxi Medical University (2023-E320-01). During the follow-up, informed consent was obtained orally from individual participants included in the study, and the investigator explained the purpose of the study to the patient or caregiver. Participants were also made aware of their right to withdraw at any time without penalty or prejudice to their future care, a principle that was strictly adhered to throughout the study period. In addition, participants who completed the survey received a complimentary disease knowledge resource as a token of appreciation and compensation for their participation, All participants’ information was confidential, and each patient was assigned an ID to keep the study data and results anonymous.

## Results

### Patients’ Baseline Characteristics

**Figure 1.**
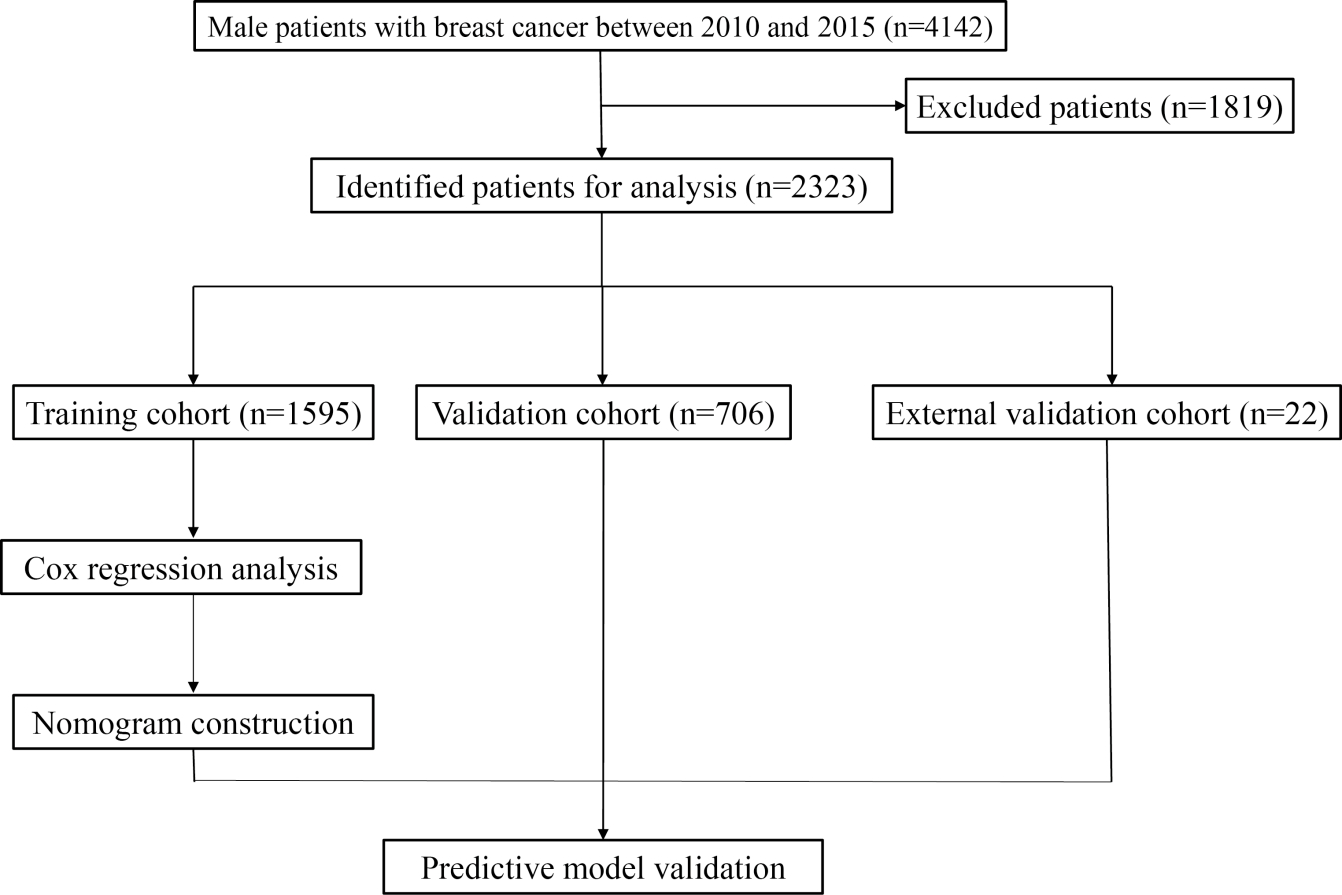
Flow chart for inclusion and partition of patients.

Figure 1 depicts the screening procedure in the SEER database. In accordance with the inclusion and exclusion criteria, a cohort of 2301 eligible patients with MBC was selected from the SEER database and randomly divided into a training set (n=1595) and an internal validation set (n=706). A total of 22 patients with MBC were chosen from the institution to serve as an external validation set. Significant variations in age were observed among the 3 groups in relation to demographic characteristics (*P*=.01). The proportion of older men in the SEER database (training set: 1180/1595, 74%; internal validation set: 505/706, 71.5%) was substantially greater than that in the external validation set (9/22, 41%). Significant differences were found in chemotherapy, lung metastasis, breast subtype, and HER2 status among the 3 groups (all *P*<.05). The proportion of men with breast cancer who received chemotherapy was higher in the external validation set (17/22, 77%) than in the SEER database (training set: 601/1595, 37.7%; internal validation set: 257/706, 36.4%). The incidence of lung metastasis in patients with MBC in the external validation set (3/22, 14%) was higher than that in the SEER database (training set: 49/1595, 3.1%; internal validation set: 24/706, 3.4%). There was a high prevalence of luminal A among men, with rates of 86.7% (1379/1595) and 85% (607/706) in the training set and internal validation set, respectively, as well as 41% (9/22) in the external validation set. A notable detail is that a significant portion of the total population exhibited a HER2-negative status accounting for 88.6% (1413/1595) in the training set, 87.5% (618/706) in the internal validation set, and 59% (13/22) in the external validation set. Table 1 displays the demographic and clinicopathological characteristics.

**Table 1.** Demographics and clinicopathologic characteristics of male breast cancer.

Variables	Total (n=2323)	Training set (n=1595)	Internal validation set (n=706)	External validation set (n=22)	*P* value
Marital status, n (%)					.10^a^
Unmarried	797 (34.3)	544 (34.1)	250 (35.4)	3 (14)	
Married	1526 (65.7)	1051 (65.9)	456 (64.6)	19 (86)	
Age, n (%)					.01^a^
≤60 years	629 (27.1)	415 (26)	201 (28.5)	13 (59)	
>60 years	1694 (72.9)	1180 (74)	505 (71.5)	9 (41)	
T^b^ stage, n (%)					.07^c^
T_0_	39 (1.7)	25 (1.6)	11 (1.6)	3 (14)	
T_1_	1047 (45.1)	717 (45)	323 (45.8)	7 (32)	
T_2_	971 (41.8)	675 (42.3)	285 (40.4)	11 (50)	
T_3_	70 (3)	46 (2.9)	23 (3.3)	1 (5)	
T_4_	196 (8.4)	132 (8.3)	64 (9.1)	0 (0)	
N stage, n (%)^d^					.30^c^
N_0_	1314 (56.6)	896 (56.2)	409 (57.9)	9 (41)	
N_1_	704 (30.3)	488 (30.6)	205 (29)	11 (50)	
N_2_	188 (8.1)	132 (8.3)	56 (7.9)	0 (0)	
N_3_	117 (5)	79 (5)	36 (5.1)	2 (9)	
M^e^ stage, n (%)					.53^a^
M_0_	2131 (91.7)	1466 (91.9)	646 (91.5)	19 (86)	
M_1_	192 (8.3)	129 (8.1)	60 (8.5)	3 (14)	
Clinical stage, n (%)					.79^c^
0	1 (0)	1 (0.1)	0 (0)	0 (0)	
I	733 (31.6)	496 (31.1)	232 (32.9)	5 (23)	
II	1020 (43.9)	704 (44.1)	304 (43.1)	12 (55)	
III	377 (16.2)	265 (16.6)	110 (15.6)	2 (9)	
IV	192 (8.3)	129 (8.1)	60 (8.5)	3 (14)	
Laterality, n (%)					.93^c^
Right	1073 (46.2)	731 (45.8)	331 (46.9)	11 (50)	
Left	1245 (53.6)	860 (53.9)	374 (53)	11 (50)	
Bilateral	5 (0.2)	4 (0.2)	1 (0.1)	0 (0)	
Surgery, n (%)					.65^a^
Yes	2119 (91.2)	1449 (90.8)	650 (92.1)	20 (91)	
No	204 (8.8)	146 (9.2)	56 (7.9)	2 (9)	
Radiation, n (%)					.37^a^
Yes	747 (32.2)	515 (32.3)	228 (32.3)	4 (18)	
No	1576 (67.8)	1080 (67.7)	478 (67.7)	18 (82)	
Chemotherapy, n (%)					<.001^a^
Yes	875 (37.7)	601 (37.7)	257 (36.4)	17 (77)	
No	1448 (62.3)	994 (62.3)	449 (63.6)	5 (23)	
Bone metastasis, n (%)					.59^c^
Yes	133 (5.7)	89 (5.6)	42 (5.9)	2 (9)	
No	2187 (94.1)	1503 (94.2)	664 (94.1)	20 (91)	
Unknown	3 (0.1)	3 (0.2)	0 (0)	0 (0)	
Brain metastasis, n (%)					.49^c^
Yes	14 (0.6)	12 (0.8)	2 (0.3)	0 (0)	
No	2302 (99.1)	1577 (98.9)	703 (99.6)	22 (100)	
Unknown	7 (0.3)	6 (0.4)	1 (0.1)	0 (0)	
Liver metastasis, n (%)					.42^c^
Yes	24 (1)	15 (0.9)	9 (1.3)	0 (0)	
No	2293 (98.7)	1574 (98.7)	697 (98.7)	22 (100)	
Unknown	6 (0.3)	6 (0.4)	0 (0)	0 (0)	
Lung metastasis, n (%)					.04^a^
Yes	76 (3.3)	49 (3.1)	24 (3.4)	3 (14)	
No	2240 (96.4)	1539 (96.5)	682 (96.6)	19 (86)	
Unknown	7 (0.3)	7 (0.4)	0 (0)	0 (0)	
Breast subtype, n (%)					<.001^a^
Luminal A	1995 (85.9)	1379 (86.5)	607 (86)	9 (41)	
Luminal B	260 (11.2)	168 (10.5)	82 (11.6)	10 (45)	
HER2^f^ -positive	22 (0.9)	14 (0.9)	6 (0.8)	2 (9)	
Triple-negative	46 (2)	34 (2.1)	11 (1.6)	1 (5)	
Estrogen receptor status, n (%)					.14^a^
Negative	72 (3.1)	52 (3.3)	18 (2.5)	2 (9)	
Positive	2251 (96.9)	1543 (96.7)	688 (97.5)	20 (91)	
Progesterone receptor status, n (%)					.06^a^
Negative	224 (9.6)	167 (10.5)	54 (7.6)	3 (14)	
Positive	2099 (90.4)	1428 (89.5)	652 (92.4)	19 (86)	
HER2 status, n (%)					.01^a^
Negative	2044 (88)	1413 (88.6)	618 (87.5)	13 (59)	
Positive	279 (12)	182 (11.4)	88 (12.5)	9 (41)	

a Chi-square test was performed.

b T: tumor.

c Fisher precision probability test was performed.

d N: lymph nodes.

e M: metastasis.

f HER2: human epidermal growth factor receptor 2.

The data of 22 male patients diagnosed with BC in the study hospital were collected. Statistically significant differences were observed between the two groups in terms of age, T stage, chemotherapy, breast subtype, and HER2 status (*P*<.05). In the unit set, ≤60 years old, T_2_ stage, receiving chemotherapy, luminal B, and HER2-positive status accounted for a greater proportion of patients with MBC. The clinicopathological characteristics of the SEER set and the unit set are provided in Multimedia Appendix 1.

### Univariate and Multivariate Cox Regression Analysis

Cox regression risk analysis was applied to conduct univariate and multivariate survival analysis for the patients with MBC in the training set. The findings indicated that age (hazard ratio [HR] 1.89, 95% CI 1.50‐2.38), marital status (HR 0.75, 95% CI 0.63‐0.89), T stage (HR 1.17, 95% CI 1.05‐1.29), clinical grade (HR 1.41, 95% CI 1.15‐1.74), surgery (HR 0.38, 95% CI 0.29‐0.51), chemotherapy (HR 0.62, 95% CI 0.50‐0.75), and HER2 status (HR 2.68, 95% CI 1.20‐5.98) were risk variables for the survival of patients with MBC (all *P*<.05; Table 2).

**Table 2.** Univariate and multivariate analysis of male breast cancer risk factors in the training set.

Variable	Univariate		Multivariate	
	HR^a^ (95% CI)	*P* value	HR (95% CI)	*P* value
Marital status	0.71 (0.60‐0.84)	<.001	0.75 (0.63‐0.89)	.01
Age	1.74 (1.39‐2.16)	<.001	1.89 (1.50‐2.38)	<.001
T^b^ stage	1.45 (1.33‐1.58)	<.001	1.17 (1.05‐1.29)	.04
N^c^ stage	1.23 (1.12‐1.35)	<.001	1.03 (0.90‐1.17)	.67
M^d^ stage	5.19 (4.18‐6.45)	<.001	1.08 (0.61‐1.91)	.81
Clinical stage	1.86 (1.70‐2.04)	<.001	1.41 (1.15‐1.74)	.01
Laterality	1.00 (0.85‐1.19)	.99	—^e^	—
Surgery	0.18 (0.15‐0.22)	<.001	0.38 (0.29‐0.51)	<.001
Radiation	0.98 (0.82‐1.18)	.84	—	—
Chemotherapy	0.78 (0.65‐0.93)	.006	0.62 (0.50‐0.75)	<.001
Bone metastasis	0.18 (0.14‐0.24)	<.001	0.71 (0.46‐1.10)	.12
Brain metastasis	0.21 (0.09‐0.49)	<.001	0.53 (0.24‐1.17)	.12
Liver metastasis	0.20 (0.10‐0.41)	<.001	0.91 (0.47‐1.80)	.79
Lung metastasis	0.25 (0.17‐0.35)	<.001	0.98 (0.69‐1.40)	.93
Breast subtype	1.16 (0.93‐1.44)	.19	2.03 (0.95‐4.30)	.07
Estrogen receptor status	0.44 (0.29‐0.65)	<.001	1.86 (0.45‐7.76)	.39
Progesterone receptor status	0.64 (0.50‐0.83)	<.001	0.91 (0.67‐1.24)	.56
HER2^f^ status	1.31 (1.02‐1.68)	.03	2.68 (1.20‐5.98)	.02

a HR: hazard ratio.

b T: tumor.

c N: lymph node.

d M: metastasis.

e Variables that were not significant in the univariate analysis do not have specific data in the multivariate analysis.

f HER2: human epidermal growth factor receptor 2.

### Construction and Validation of Nomogram

**Figure 2.**
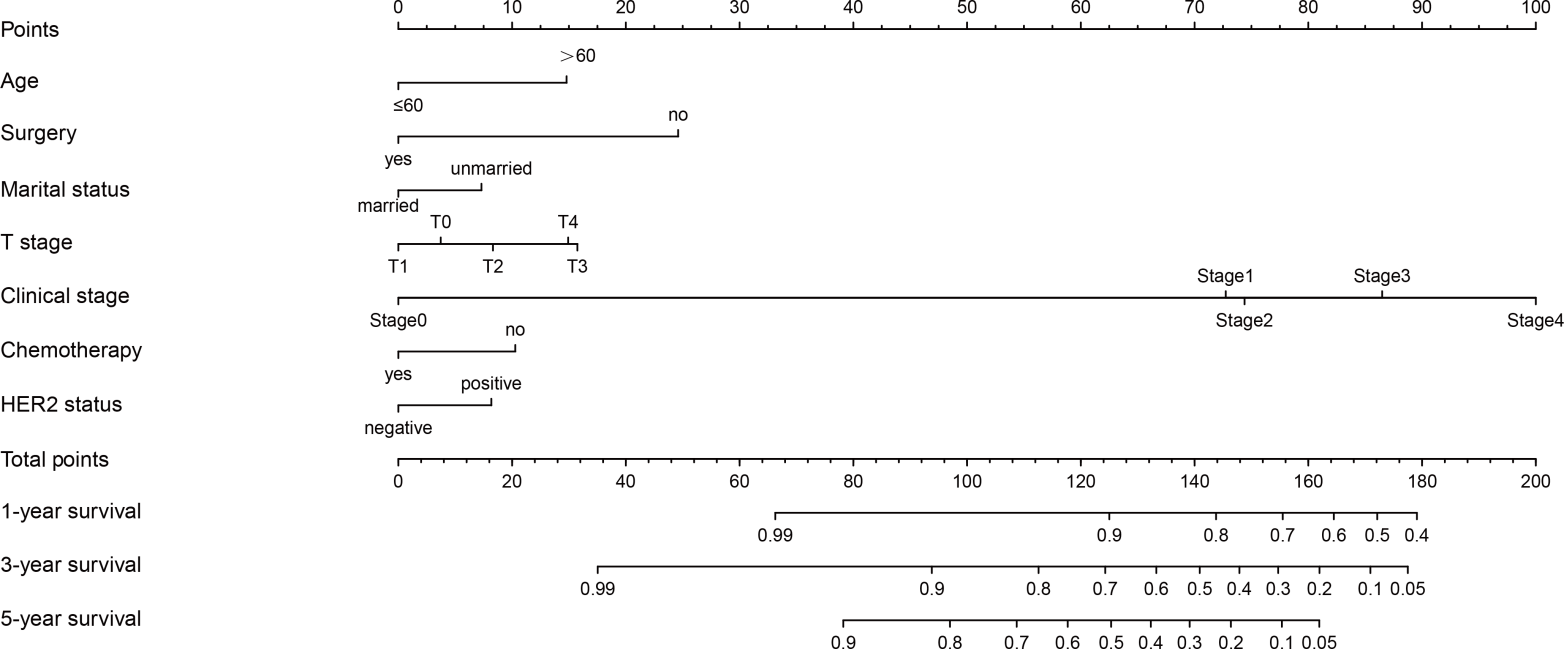
Nomogram for predicting 1-, 3- and 5-year overall survival in patients with male breast cancer. HER2: human epidermal growth factor receptor 2; T: tumor.

The construction of a nomogram for the overall survival prognosis of MBC was based on the results of the Cox regression analysis conducted on the training set. This analysis identified 7 variables that were subsequently used in the development of the nomogram (Figure 2). The nomogram demonstrated that clinical stage and surgery were the primary vital risk variables that affect the survival outcomes of patients with MBC. The total score could predict the 1-, 3-, and 5-year survival rates of patients with MBC by summing the scores of each variable.

**Figure 3.**
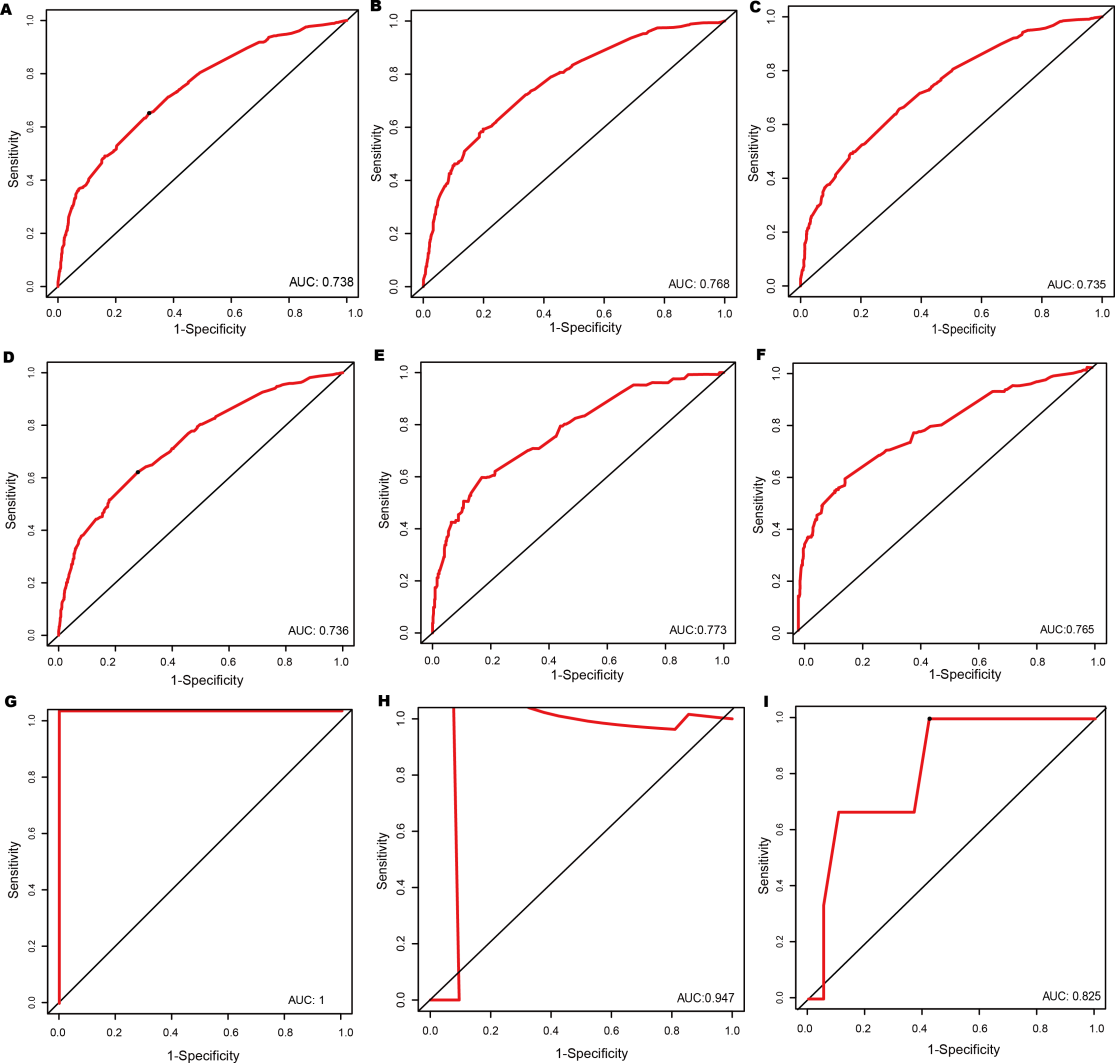
Receiver operating characteristic curve of prediction of 1-, 3-, and 5-year survival in the training set (A-C), internal validation set (D-F), and external validation set (G–I). AUC: area under the curve.

The discrimination ability of the nomogram was evaluated in this study by using the ROC curve and the C-index. The area under the curve values of the nomogram at 1-, 3-, and 5-year survival probabilities had excellent discrimination efficacy in the training set (Figure 3A-C). The area under the curve values in the internal validation set were 0.736, 0.773, and 0.765 (Figure 3D-F), and the external validation set values were 1, 0.947, and 0.825 (Figure 3G-I). The C-index of the training set was determined using the bootstrap method, and the C-index of the external validation set was 0.72, 0.747, and 0.981 for the at 1-, 3-, and 5-year survival, respectively, indicating that the nomogram exhibited a favorable discriminatory capability in the American and Chinese populations.

**Figure 4.**
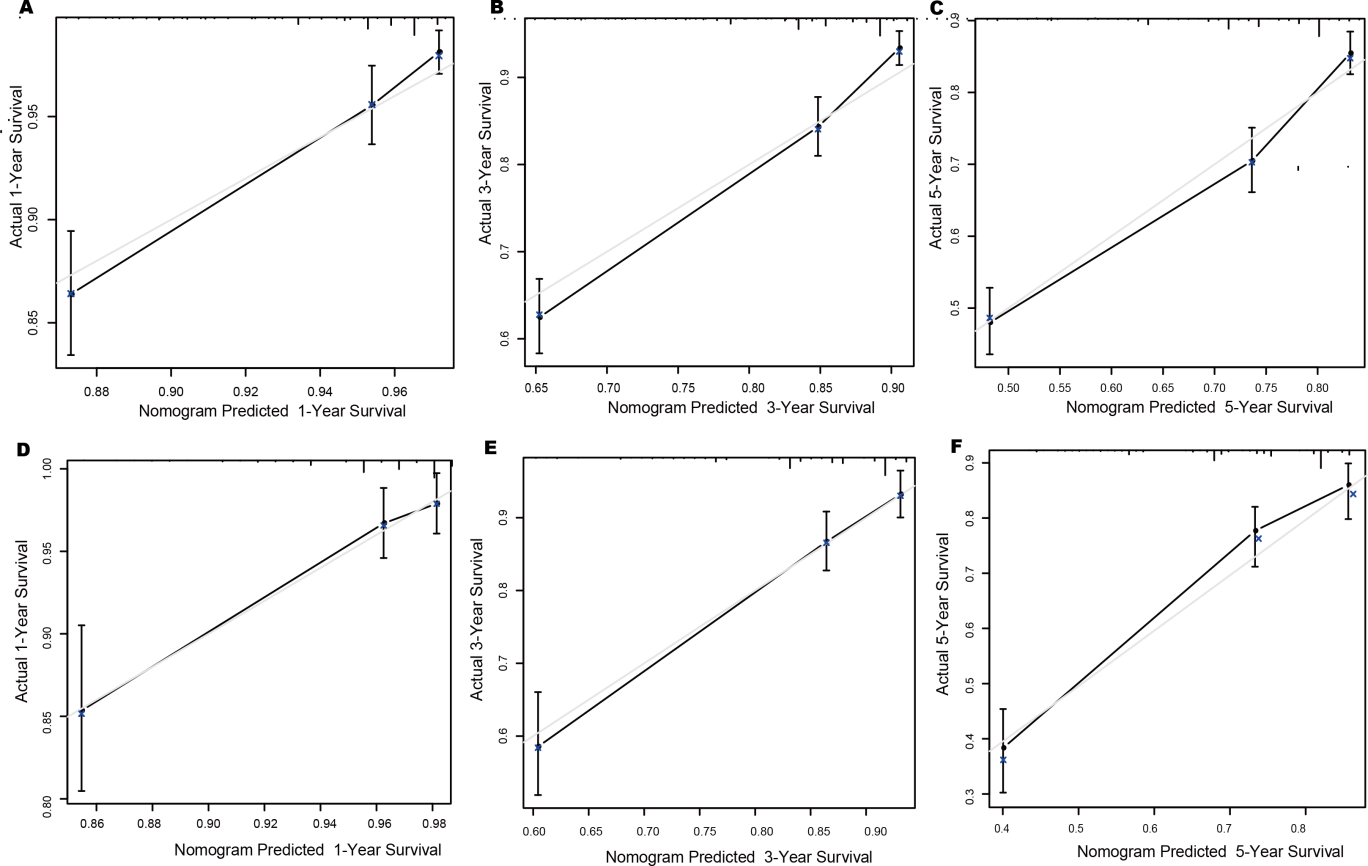
Calibration curve of 1-, 3-, and 5-year overall survival in the training set (A-C) and internal validation set (D-F). The errors bars represent the 95% CI of these estimates.

The calibration curves were used to evaluate the consistency of the nomogram. The findings indicated a high degree of uniformity between the predicted and observed probabilities of survival in the training set (Figure 4A-C) and internal validation set (Figure 4D-F).

**Figure 5.**
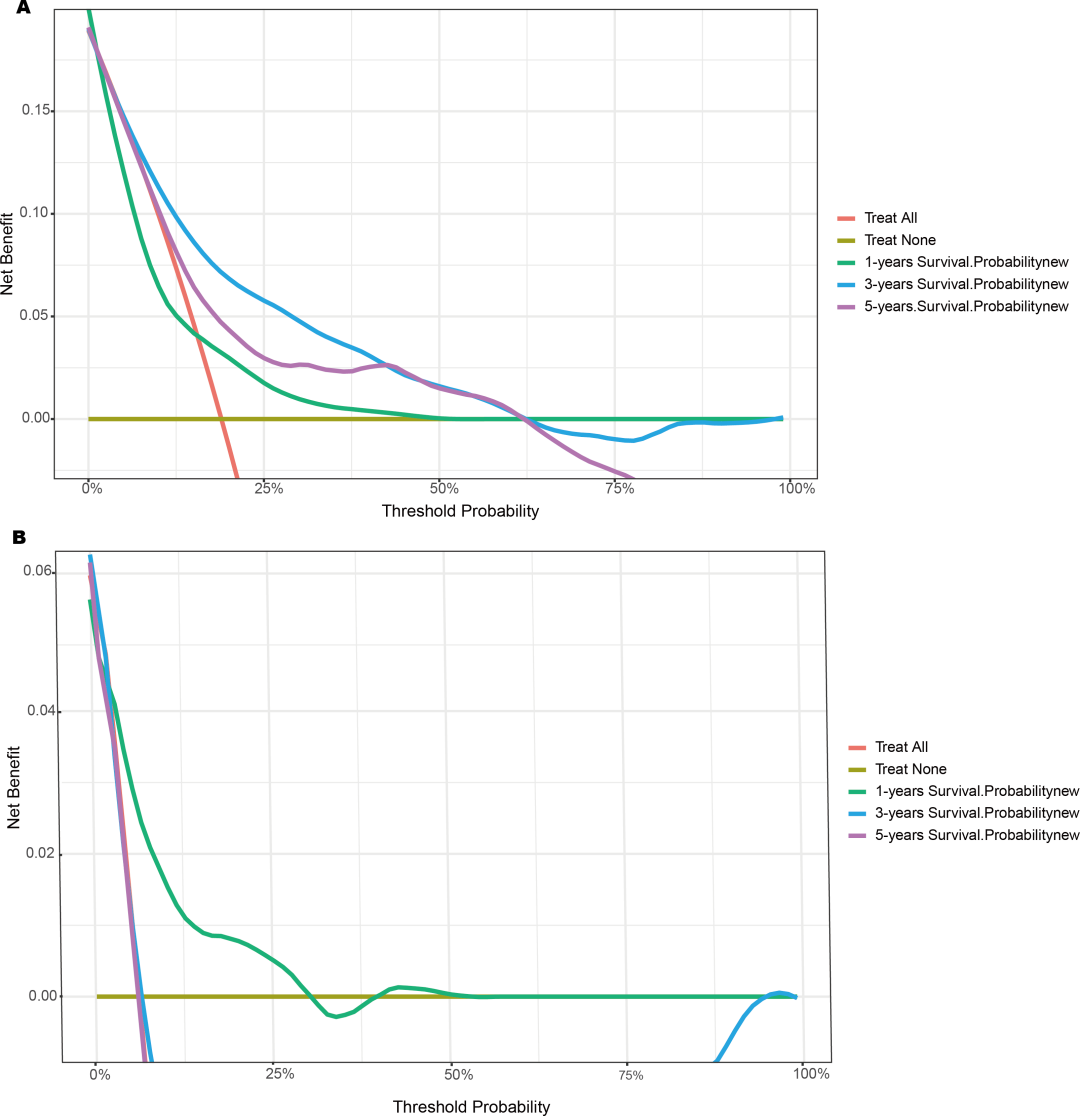
Decision curve analysis of 1-, 3- and 5-year survival in the training set (A) and the internal validation set (B).

The DCA curve demonstrated that the nomogram exhibited superior performance in terms of net clinical benefit and predictive accuracy for 3- and 5-year survival outcomes in the training set (Figure 5A) and internal validation set (Figure 5B).

### Nomogram Prediction Score Risk Stratification

**Figure 6.**
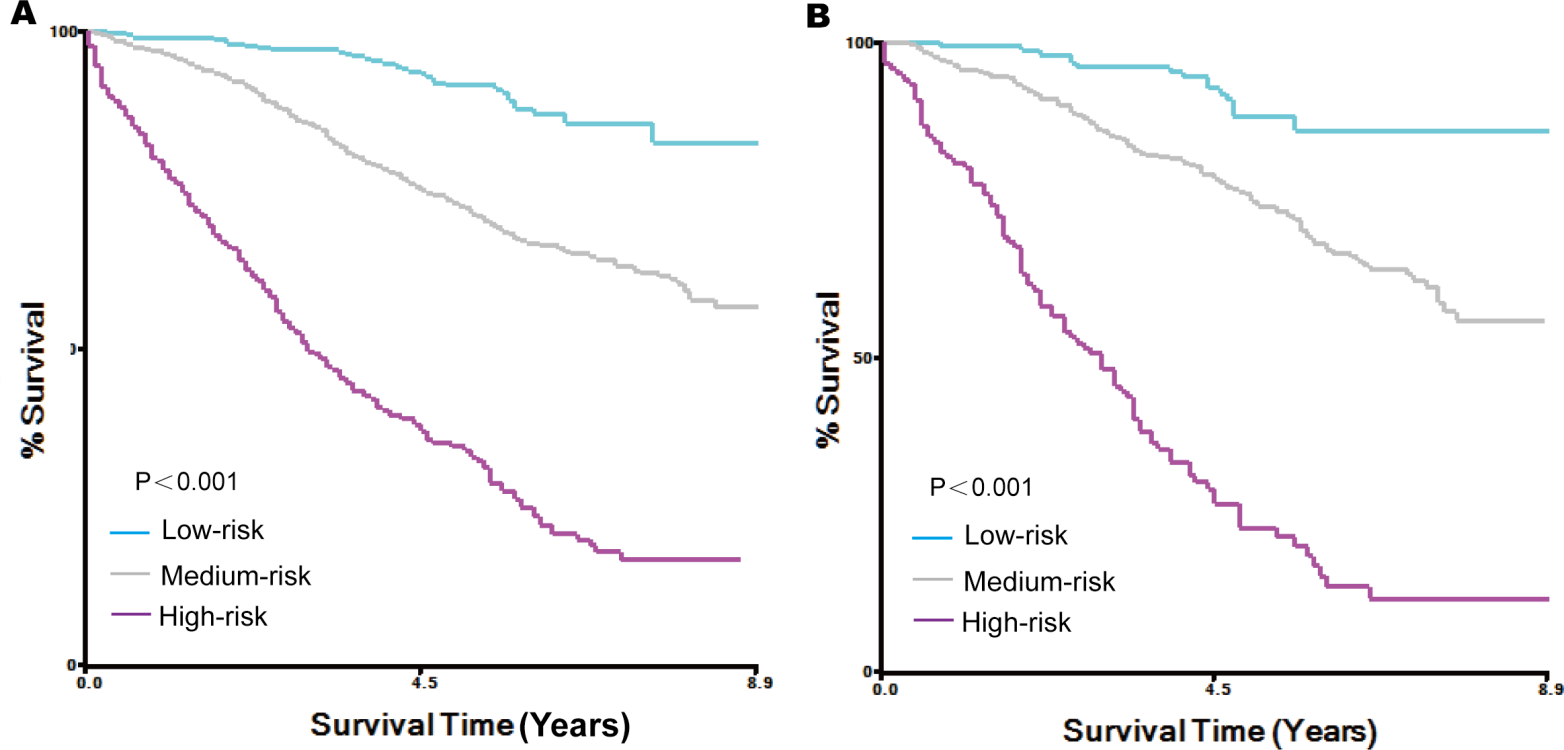
Analysis of survival based on risk stratification. Kaplan-Meier curve for patients categorized as low-risk, medium-risk, or high-risk in the training set (A) and internal validation set (B).

Finally, risk stratification was conducted by calculating the nomogram total score of each individual in the training set (Table 3). After the cut-off values were determined using X-tile software, all patients with MBC were divided into 3 groups: low-risk group (points≤93), medium-risk group (93<points≤117), and high-risk group (points>117). The survival curves of each risk group were depicted using the Kaplan-Meier model in the training set (Figure 6A) and internal validation set (Figure 6B). A log-rank test was used to compare the differences between the groups to assess the accuracy of risk stratification on the basis of the nomogram score.

**Table 3.** Nomogram score of male breast cancer survival.

Variable	Points
Age	
≤60 years	0
>60 years	15
Surgery	
Yes	0
No	25
Marital status	
Unmarried	7
Married	0
T^a^ stage	
T_0_	4
T_1_	0
T_2_	8
T_3_	16
T_4_	15
Clinical stage	
0	0
I	73
II	74
III	86
IV	100
Chemotherapy	
Yes	0
No	10
HER2^b^ status	
Positive	8
Negative	0

a T: tumor.

b HER2: human epidermal growth factor receptor 2.

## Discussion

### Principal Findings

In this study, we developed a nomogram to predict survival in MBC based on the SEER database and validated it using both an internal validation dataset from this database and an external validation cohort from a single center. We identified 7 independent risk factors and incorporated them into the nomogram to predict the survival of patients with MBC. The results of both internal and external validation demonstrated that the nomogram exhibited good accuracy and discriminative power, confirming the robustness of the prediction model.

MBC has a low incidence and is a rare malignancy [24]. However, MBC exhibits a delayed onset, presents as a more advanced disease, and has a less unfavorable prognosis than female BC [25]. Due to its rarity, MBC is often overlooked in clinical practice. The assessment of prognosis in MBC holds considerable importance for facilitating the implementation of comprehensive treatment strategies. This study used the clinical data of 1595 patients with MBC from the SEER database to establish a nomogram for prognosticating the survival in MBC. The bootstrap method was used for internal validation, and external validation was performed in the hospital cohort. The ROC curve, C-index, and calibration curve were used to assess the discrimination and reliability of the nomogram. Additionally, the clinical benefit and application value of the nomogram were evaluated using the DCA curve. The findings demonstrated that the nomogram can accurately and individually predict the survival outcomes of patients with MBC. This predictive tool holds the potential for informing clinical decision-making and guiding the development of appropriate diagnostic and therapeutic strategies.

Previous studies analyzed the influencing factors of MBC survival by using univariate and multivariate Cox proportional hazards regression models [26,27]. Compared with traditional multivariate regression, least absolute shrinkage and selection operator (LASSO) regression is widely regarded as a superior approach for variable selection owing to its ability to mitigate model complexity, minimizing overfitting by incorporating a loss function or a penalty term into the objective function. In this study, the LASSO regression algorithm identified 7 variables (age, surgery, marital status, T stage, clinical stage, chemotherapy, and HER2 status) as factors that are associated with the prognosis of MBC. This detail has also been recognized in other studies [9,28]. Based on the aforementioned variables, a nomogram prediction model that significantly enhanced the clinical applicability within various clinical scenarios was developed. The nomogram exhibited good discrimination, consistency, and clinical validity in the training set and validation set. It may guide clinical decision-making for these patients more effectively.

Age was identified as a significant risk factor for the survival of patients with MBC in the nomogram, and individuals aged over 60 years had higher mortality, consistent with prior studies [28,29]. This finding may be related to the presence of more comorbidities in older patients [28]. Surgery and chemotherapy are essential for determining the prognosis of patients with MBC who are undergoing treatment, a finding that is similar to that of previous studies [30-32]. A recent study conducted by Wang et al [33] indicated that patients diagnosed with MBC who received surgery or chemotherapy exhibited a more favorable prognosis than individuals who did not undergo these treatments. The prognostic significance of marital status was observed, with unmarried patients exhibiting a poorer prognosis [34-36]. The reason for this result may be that unmarried patients with MBC experience more significant psychological distress, including feelings of sadness and anxiety, compared to married patients [37], and they may demonstrate greater adherence to treatment regimens [38], which could improve cancer management. Additionally, this study provided evidence to support the notion that the T stage and clinical stage are prognostic indicators for MBC [39,40]. Among the 7 parameters in the nomogram, the clinical stage showed the most significant influence on overall survival, and patients with stages III and IV MBC had the worst prognosis. One study in Serbia showed that low initial disease stage and low tumor grade are independent predictors of a good prognosis in patients with MBC [41]. In addition, having a HER2-positive tumor is widely acknowledged as a significant prognostic factor for MBC, and this observation has been corroborated by other investigations [42-44].

In this study, the average age of onset of Chinese patients with MBC may be younger than that of patients in the SEER database, which is similar to the onset characteristics of female patients with BC [45]. In addition, the hospital exhibited a higher proportion of patients in the early T stage than the SEER database. The proportion of patients undergoing chemotherapy was significantly greater than that in the SEER database, contributing to the favorable prognosis observed in Chinese patients with MBC.

Constructing a nomogram for the survival of patients with MBC can be beneficial for medical staff to intuitively analyze the weight of risk factors and the corresponding survival probabilities of patients. These survival probabilities can be used as a basis for stratification. The patients were classified into 3 groups: low-, medium-, and high-risk. For example, a patient with MBC that is over 60 years old, is married, has undergone surgery and chemotherapy, has a T grade of T_2_, has a tumor stage of II, and has a HER2-positive tumor, would have a total score of approximately 105, belonging to the medium-risk group for survival. Therefore, the medical staff should take relevant measures to timely manage and improve the prognosis of this patient. In clinical practice, the proposed model can be used to determine and evaluate the survival rate and prognosis of patients with MBC. This approach aims to provide personalized and accurate survival rate and prognosis and then develop targeted clinical decisions for patients with MBC.

### Strength and Limitations

Our study has the following strengths. First, the existing prognostic models for BC have a focus on female BC, and little focus has been given to MBC. The study aims to develop a prognostic model specifically for this group. Second, the SEER database included a large and diverse cohort, ensuring robust and representative results. In addition, external validation using datasets from our own hospital further confirmed the model’s accuracy and generalizability.

However, this study has some limitations. First, as a retrospective study, it is subject to selection bias. Second, important variables, such as endocrine therapy, BMI, and the cellular proliferation marker Ki-67, are not included in the SEER database, which may limit the accuracy and effectiveness of the nomogram. Finally, the external validation sample size in this study was limited, including only retrospective data from a single health care institution, and the predictive ability of the model for the Chinese population needs to be further verified using a large sample of data.

### Future Directions

Future studies should consider incorporating data from multicenter cohorts to increase the sample size, thereby enhancing the accuracy and generalizability of survival prediction models for MBC. By collecting data from diverse geographic locations, researchers can ensure that the model captures a broader range of clinical variables, improving its robustness and applicability. Additionally, prospective cohort studies should be conducted to externally validate the model in real-world clinical settings and assess its practical utility in daily clinical decision-making for MBC. Furthermore, integrating additional datasets that include critical variables, such as BMI and the cellular proliferation marker Ki-67, would further strengthen the model’s predictive power.

### Conclusion

In summary, a nomogram was developed using 7 variables to predict the prognosis of patients with MBC, and age, surgery, marital status, T stage, clinical stage, and HER2 status were identified as independent risk factors for predicting the survival of patients with MBC. Internal and external verifications proved that the model has good accuracy and reliability. Thus, it could serve as an accurate and individualized tool that clinicians could use for decision-making.

## Supplementary material

10.2196/54625 Multimedia Appendix 1 Clinicopathological characteristics of the Surveillance, Epidemiology, and End Results (SEER) dataset and our dataset.

10.2196/54625 Checklist 1 Transparent Reporting of a Multivariable Prediction Model for Individual Prognosis or Diagnosis (TRIPOD) checklist for prediction model development and validation.

## Abbreviations

BC: breast cancer
C-index: concordance index
DCA: decision curve analysis
HER2: human epidermal growth factor receptor 2
LASSO: least absolute shrinkage and selection operator
MBC: male breast cancer
ROC: receiver operating characteristic
SEER: Surveillance, Epidemiology, and End Results
TNM: tumor, extent of spread to the lymph nodes, and presence of metastasis

## References

[1] Wang W, Xu X, Tian B (2019). Clinical features of patients with male breast cancer in Shanxi province of China from 2007 to 2016. J Investig Med.

[2] Abdelwahab Yousef AJ (2017). Male breast cancer: epidemiology and risk factors. Semin Oncol.

[3] Gucalp A, Traina TA, Eisner JR (2019). Male breast cancer: a disease distinct from female breast cancer. Breast Cancer Res Treat.

[4] Song AL, Ou JH (2023). Clinical practice guideline for diagnosis and treatment of male breast cancer in China. Chin J Pract Surg.

[5] Konduri S, Singh M, Bobustuc G, Rovin R, Kassam A (2020). Epidemiology of male breast cancer. Breast.

[6] Skop M, Lorentz J, Jassi M, Vesprini D, Einstein G (2018). “Guys don’t have breasts”: the lived experience of men who have BRCA gene mutations and are at risk for male breast cancer. Am J Mens Health.

[7] Rojas K, Stuckey A (2016). Breast cancer epidemiology and risk factors. Clin Obstet Gynecol.

[8] Lin AP, Huang TW, Tam KW (2021). Treatment of male breast cancer: meta-analysis of real-world evidence. Br J Surg.

[9] Leone JP, Zwenger AO, Iturbe J (2016). Prognostic factors in male breast cancer: a population-based study. Breast Cancer Res Treat.

[10] Giordano SH (2018). Breast cancer in men. N Engl J Med.

[11] Rice TW, Ishwaran H, Ferguson MK, Blackstone EH, Goldstraw P (2017). Cancer of the esophagus and esophagogastric junction: an eighth edition staging primer. J Thorac Oncol.

[12] Wang J, Zhou J, Liu L, Wu SG (2022). Stage-specific survival in breast cancer in chinese and white women: comparative data analysis. JMIR Public Health Surveill.

[13] Yang SP, Su HL, Chen XB (2021). Long-term survival among histological subtypes in advanced epithelial ovarian cancer: population-based study using the surveillance, epidemiology, and end results database. JMIR Public Health Surveill.

[14] Donegan WL, Redlich PN, Lang PJ, Gall MT (1998). Carcinoma of the breast in males: a multiinstitutional survey. Cancer.

[15] Anderson WF, Jatoi I, Tse J, Rosenberg PS (2010). Male breast cancer: a population-based comparison with female breast cancer. J Clin Oncol.

[16] Bray F, Ferlay J, Soerjomataram I, Siegel RL, Torre LA, Jemal A (2018). Global cancer statistics 2018: GLOBOCAN estimates of incidence and mortality worldwide for 36 cancers in 185 countries. CA Cancer J Clin.

[17] Balachandran VP, Gonen M, Smith JJ, DeMatteo RP (2015). Nomograms in oncology: more than meets the eye. Lancet Oncol.

[18] Palzer EF, Vempati S, Helgeson ES, Matas AJ (2020). Long-term living kidney donor risk: a web-based calculator. J Am Soc Nephrol.

[19] Gao Y, Li S, Jin Y (2022). An assessment of the predictive performance of current machine learning-based breast cancer risk prediction models: systematic review. JMIR Public Health Surveill.

[20] Yang X, Qiu H, Wang L, Wang X (2023). Predicting colorectal cancer survival using time-to-event machine learning: retrospective cohort study. J Med Internet Res.

[21] Wu J, Zhang H, Li L (2020). A nomogram for predicting overall survival in patients with low-grade endometrial stromal sarcoma: a population-based analysis. Cancer Commun (Lond).

[22] Huang X, Luo Z, Liang W (2022). Survival nomogram for young breast cancer patients based on the SEER database and an external validation cohort. Ann Surg Oncol.

[23] Wang Z, Gao L, Guo X (2019). Development and validation of a nomogram with an autophagy-related gene signature for predicting survival in patients with glioblastoma. Aging (Albany NY).

[24] Chen W, Zheng R, Baade PD (2016). Cancer statistics in China, 2015. CA Cancer J Clin.

[25] Malani AK (2007). Male breast cancer: a different disease than female breast cancer?. South Med J.

[26] Zhao J, Wang B, Zhao J, Mao Y, Liu J, Yang Y (2020). Male breast cancer: a closer look at patient and tumor characteristics and factors associated with survival. Thorac Cancer.

[27] Jylling AMB, Jensen V, Lelkaitis G, Christiansen P, Nielsen SS, Lautrup MD (2020). Male breast cancer: clinicopathological characterization of a national Danish cohort 1980-2009. Breast Cancer (Auckl).

[28] Yadav S, Karam D, Bin Riaz I (2020). Male breast cancer in the United States: treatment patterns and prognostic factors in the 21st century. Cancer.

[29] Khan NAJ, Tirona M (2021). An updated review of epidemiology, risk factors, and management of male breast cancer. Med Oncol.

[30] Singh R, Cao L, Sarode AL, Kharouta M, Shenk R, Miller ME (2023). Trends in surgery and survival for T1-T2 male breast cancer: a study from the National Cancer Database. Am J Surg.

[31] Zhang J, Jiang H, Zhang J (2021). Effectiveness and safety of pegylated liposomal doxorubicin versus epirubicin as neoadjuvant or adjuvant chemotherapy for breast cancer: a real-world study. BMC Cancer.

[32] Pan H, Zhang K, Wang M, Ling L, Wang S, Zhou W (2020). The effect of chemotherapy on survival in patients with nonmetastatic male breast cancer: a population-based observational study. Cancer.

[33] Wang J, Sun Y, Qu J (2019). Survival analysis for male ductal and lobular breast cancer patients with different stages. Future Oncol.

[34] Adekolujo OS, Tadisina S, Koduru U, Gernand J, Smith SJ, Kakarala RR (2017). Impact of marital status on tumor stage at diagnosis and on survival in male breast cancer. Am J Mens Health.

[35] Liu L, Chi YY, Wang AA, Luo Y (2018). Marital status and survival of patients with hormone receptor-positive male breast cancer: a surveillance, epidemiology, and end results (SEER) population-based study. Med Sci Monit.

[36] Krajc K, Miroševič Š, Sajovic J (2023). Marital status and survival in cancer patients: a systematic review and meta-analysis. Cancer Med.

[37] Goldzweig G, Andritsch E, Hubert A (2010). Psychological distress among male patients and male spouses: what do oncologists need to know?. Ann Oncol.

[38] Tran BX, Fleming M, Do HP, Nguyen LH, Latkin CA (2018). Quality of life improvement, social stigma and antiretroviral treatment adherence: implications for long-term HIV/AIDS care. AIDS Care.

[39] Methamem M, Ghadhab I, Hidar S, Briki R (2020). Breast cancer in men: a serie of 45 cases and literature review. Pan Afr Med J.

[40] André S, Pereira T, Silva F (2019). Male breast cancer: specific biological characteristics and survival in a Portuguese cohort. Mol Clin Oncol.

[41] Sipetic-Grujicic SB, Murtezani ZH, Neskovic-Konstatinovic ZB (2014). Multivariate analysis of prognostic factors in male breast cancer in Serbia. Asian Pac J Cancer Prev.

[42] Sokmen FC (2020). Prognostic factors in male breast cancer: a single centre experience. J Coll Physicians Surg Pak.

[43] Abreu MH, Afonso N, Abreu PH (2016). Male breast cancer: looking for better prognostic subgroups. Breast.

[44] Leone J, Freedman RA, Lin NU (2021). Tumor subtypes and survival in male breast cancer. Breast Cancer Res Treat.

[45] Fan L, Strasser-Weippl K, Li JJ (2014). Breast cancer in China. Lancet Oncol.

