# Supplementary material for: Predicting Overall Survival in Patients with Male Breast Cancer: Nomogram Development and External Validation Study
Source: JMIR Cancer. 2025 Mar 4;11:e54625. doi: 10.2196/54625 (PMC11896567; doi:10.2196/54625)
Supplement: Multimedia Appendix 1 [file cancer-v11-e54625-s001.docx]

Table S1 Clinicopathological characteristics of the SEER set and our set

| Variables | Total (n = 2323) | SEER set  (n = 2301) | Our unit set  (n = 22) | *P*-value |
| --- | --- | --- | --- | --- |
| **Marital status, n (%)** | |  |  | .068 |
| Unmarried | 797 (34) | 794 (35) | 3 (14) |  |
| Married | 1526 (66) | 1507 (65) | 19 (86) |  |
| **Age, n (%)** |  |  |  | .002 |
| ≤ 60 | 629 (27) | 616 (27) | 13 (59) |  |
| > 60 | 1694 (73) | 1685 (73) | 9 (41) |  |
| **T stage, n (%)** | |  |  | .008 |
| T_0_ | 39 (2) | 36 (2) | 3 (14) |  |
| T_1_ | 1047 (45) | 1040 (45) | 7 (32) |  |
| T_2_ | 971 (42) | 960 (42) | 11 (50) |  |
| T_3_ | 70 (3) | 69 (3) | 1 (5) |  |
| T_4_ | 196 (8) | 196 (9) | 0 (0) |  |
| **N stage, n (%)** | |  |  | .079 |
| N_0_ | 1314 (57) | 1305 (57) | 9 (41) |  |
| N_1_ | 704 (30) | 693 (30) | 11 (50) |  |
| N_2_ | 188 (8) | 188 (8) | 0 (0) |  |
| N_3_ | 117 (5) | 115 (5) | 2 (9) |  |
| **M stage, n (%)** | |  |  | .42 |
| M_0_ | 2131 (92) | 2112 (92) | 19 (86) |  |
| M_1_ | 192 (8) | 189 (8) | 3 (14) |  |
| **Clinical stage, n (%)** | |  |  | .445 |
| 0 | 1 (0) | 1 (0) | 0 (0) |  |
| Ⅰ | 733 (32) | 728 (32) | 5 (23) |  |
| Ⅱ | 1020 (44) | 1008 (44) | 12 (55) |  |
| Ⅲ | 377 (16) | 375 (16) | 2 (9) |  |
| Ⅳ | 192 (8) | 189 (8) | 3 (14) |  |
| **Laterality, n (%)** | |  |  | .839 |
| Right | 1073 (46) | 1062 (46) | 11 (50) |  |
| Left | 1245 (54) | 1234 (54) | 11 (50) |  |
| Bilateral | 5 (0) | 5 (0) | 0 (0) |  |
| **surgery, n (%)** | |  |  | 1 |
| Yes | 2119 (91) | 2099 (91) | 20 (91) |  |
| No | 204 (9) | 202 (9) | 2 (9) |  |
| **Radiation, n (%)** | |  |  | .238 |
| Yes | 747 (32) | 743 (32) | 4 (18) |  |
| No | 1576 (68) | 1558 (68) | 18 (82) |  |
| **Chemotherapy, n (%)** | |  |  | < .001 |
| Yes | 875 (38) | 858 (37) | 17 (77) |  |
| No | 1448 (62) | 1443 (63) | 5 (23) |  |
| **Bone met, n (%)** | |  |  | .38 |
| Yes | 133 (6) | 131 (6) | 2 (9) |  |
| No | 2187 (94) | 2167 (94) | 20 (91) |  |
| Unknown | 3 (0) | 3 (0) | 0 (0) |  |
| **Brain met, n (%)** | |  |  | 1 |
| Yes | 14 (1) | 14 (1) | 0 (0) |  |
| No | 2302 (99) | 2280 (99) | 22 (100) |  |
| Unknown | 7 (0) | 7 (0) | 0 (0) |  |
| **Liver met, n (%)** | |  |  | 1 |
| Yes | 24 (1) | 24 (1) | 0 (0) |  |
| No | 2293 (99) | 2271 (99) | 22 (100) |  |
| Unknown | 6 (0) | 6 (0) | 0 (0) |  |
| **Lung met, n (%)** | |  |  | .065 |
| Yes | 76 (3) | 73 (3) | 3 (14) |  |
| No | 2240 (96) | 2221 (97) | 19 (86) |  |
| Unknown | 7 (0) | 7 (0) | 0 (0) |  |
| **Breast subtype, n (%)** | |  |  | < .001 |
| Luminal A | 1995 (86) | 1986 (86) | 9 (41) |  |
| Luminal B | 260 (11) | 250 (11) | 10 (45) |  |
| Her2++ | 22 (1) | 20 (1) | 2 (9) |  |
| Triple-negative | 46 (2) | 45 (2) | 1 (5) |  |
| **ER status, n (%)** | |  |  | .147 |
| Negative | 72 (3) | 70 (3) | 2 (9) |  |
| Positive | 2251 (97) | 2231 (97) | 20 (91) |  |
| **PR status, n (%)** | |  |  | .464 |
| Negative | 224 (10) | 221 (10) | 3 (14) |  |
| Positive | 2099 (90) | 2080 (90) | 19 (86) |  |
| **HER2 status, n (%)** | |  |  | < .001 |
| Negative | 2044 (88) | 2031 (88) | 13 (59) |  |
| Positive | 279 (12) | 270 (12) | 9 (41) |  |

Notes: T, Tumor; N, Lymph node; Met, Metastasis; ER, Estrogen receptor; PR, Progesterone receptor; HER2, Human Epidermal Growth Factor Receptor 2; Met: Metastases.
